# An integrative ENCODE resource for cancer genomics

**DOI:** 10.1038/s41467-020-14743-w

**Published:** 2020-07-29

**Authors:** Jing Zhang, Donghoon Lee, Vineet Dhiman, Peng Jiang, Jie Xu, Patrick McGillivray, Hongbo Yang, Jason Liu, William Meyerson, Declan Clarke, Mengting Gu, Shantao Li, Shaoke Lou, Jinrui Xu, Lucas Lochovsky, Matthew Ung, Lijia Ma, Shan Yu, Qin Cao, Arif Harmanci, Koon-Kiu Yan, Anurag Sethi, Gamze Gürsoy, Michael Rutenberg Schoenberg, Joel Rozowsky, Jonathan Warrell, Prashant Emani, Yucheng T. Yang, Timur Galeev, Xiangmeng Kong, Shuang Liu, Xiaotong Li, Jayanth Krishnan, Yanlin Feng, Juan Carlos Rivera-Mulia, Jessica Adrian, James R Broach, Michael Bolt, Jennifer Moran, Dominic Fitzgerald, Vishnu Dileep, Tingting Liu, Shenglin Mei, Takayo Sasaki, Claudia Trevilla-Garcia, Su Wang, Yanli Wang, Chongzhi Zang, Daifeng Wang, Robert J. Klein, Michael Snyder, David M. Gilbert, Kevin Yip, Chao Cheng, Feng Yue, X. Shirley Liu, Kevin P. White, Mark Gerstein

**Affiliations:** 1 Program in Computational Biology & Bioinformatics, Yale University, New Haven, CT 06520 USA; 2 Department of Molecular Biophysics & Biochemistry, Yale University, New Haven, CT 06520 USA; 3 Department of Human Genetics, University of Chicago, Chicago, IL 60637 USA; 4 Institute for Genomics and Systems Biology, University of Chicago, Chicago, IL 60637 USA; 5 Department of Data Science, Dana-Farber Cancer Institute and Harvard T.H. Chan School of Public Health, Boston, MA 02215 USA; 6 Cancer Data Science Lab, Center for Cancer Research, National Cancer Institute, National Institutes of Health, Bethesda, MD 20892 USA; 7 Department of Biochemistry and Molecular Genetics, Feinberg School of Medicine, Northwestern University, Chicago, IL 60611 USA; 8 Robert H. Lurie Comprehensive Cancer Center of Northwestern University, Chicago, IL 60611 USA; 9 Department of Biochemistry and Molecular Biology, College of Medicine, The Pennsylvania State University, Hershey, PA 17033 USA; 10 Department of Biomedical Data Science, Department of Molecular and Systems Biology, Geisel School of Medicine at Dartmouth, Lebanon, NH 03765 USA; 11 School of Life Sciences, Westlake University, Hangzhou, Zhejiang 310024 China; 12 Department of Computer Science and Engineering, The Chinese University of Hong Kong, Shatin, Hong Kong SAR; 13 Center for Precision Health, School of Biomedical Informatics, University of Texas Health Science Center at Houston, Houston, TX 77030 USA; 14 Department of Biological Science, Florida State University, Tallahassee, FL 32306 USA; 15 Department of Biochemistry, Molecular Biology and Biophysics, University of Minnesota Medical School, Minneapolis, MN 55455 USA; 16 Department of Genetics, School of Medicine, Stanford University, Stanford, CA 94305 USA; 17 Department of Biomedical Informatics, Harvard Medical School, Boston, MA 02115 USA; 18 Center for Public Health Genomics, Department of Public Health Sciences, University of Virginia, Charlottesville, VA 22908 USA; 19 Department of Biostatistics and Medical Informatics, University of Wisconsin-Madison, Madison, WI 53726 USA; 20 Waisman Center, University of Wisconsin-Madison, Madison, WI 53705 USA; 21 Department of Genetics and Genomic Sciences, Icahn Institute for Data Science and Genomic Technology, Icahn School of Medicine at Mount Sinai, New York, NY 10029 USA; 22 Department of Medicine, Institute for Clinical and Translational Research, Baylor College of Medicine, Houston, TX 77030 USA; 23 Tempus Labs, Chicago, IL 60654 USA; 24 Department of Computer Science, Yale University, New Haven, CT 06520 USA; 25 Department of Statistics & Data Science, Yale University, New Haven, CT 06520 USA

**Keywords:** Data integration, Gene regulatory networks, Cancer genomics

## Abstract

ENCODE comprises thousands of functional genomics datasets, and the encyclopedia covers hundreds of cell types, providing a universal annotation for genome interpretation. However, for particular applications, it may be advantageous to use a customized annotation. Here, we develop such a custom annotation by leveraging advanced assays, such as eCLIP, Hi-C, and whole-genome STARR-seq on a number of data-rich ENCODE cell types. A key aspect of this annotation is comprehensive and experimentally derived networks of both transcription factors and RNA-binding proteins (TFs and RBPs). Cancer, a disease of system-wide dysregulation, is an ideal application for such a network-based annotation. Specifically, for cancer-associated cell types, we put regulators into hierarchies and measure their network change (rewiring) during oncogenesis. We also extensively survey TF-RBP crosstalk, highlighting how SUB1, a previously uncharacterized RBP, drives aberrant tumor expression and amplifies the effect of MYC, a well-known oncogenic TF. Furthermore, we show how our annotation allows us to place oncogenic transformations in the context of a broad cell space; here, many normal-to-tumor transitions move towards a stem-like state, while oncogene knockdowns show an opposing trend. Finally, we organize the resource into a coherent workflow to prioritize key elements and variants, in addition to regulators. We showcase the application of this prioritization to somatic burdening, cancer differential expression and GWAS. Targeted validations of the prioritized regulators, elements and variants using siRNA knockdowns, CRISPR-based editing, and luciferase assays demonstrate the value of the ENCODE resource.

## Abstract (web summary)

ENCODE is a resource comprising thousands of functional genomic datasets. Here, the authors present custom annotation within ENCODE for cancer, highlighting a workflow that can help prioritise key elements in oncogenesis.

## Introduction

The 2012 ENCODE release provided comprehensive functional genomics data, such as RNA-seq, histone modification and transcription factor (TF) ChIP-seq, and DNase-seq, to annotate the noncoding regions in the human genome^1^. After the release, the cancer genomics community embraced the ENCODE data, together with other functional genomic data, to study the mutational landscape and regulatory networks in cancer^2–8^.

The current release broadens the number of cell lines and considerably expands the available tissue data. It also greatly increases the depth by adding advanced assays, such as eCLIP, RAMPAGE, ChIA-PET, Hi-C, and whole-genome STARR-seq. The ENCODE encyclopedia takes advantage of the breadth of ENCODE data to provide a universal annotation across hundreds of cell types. It uniformly constructs regulatory elements using assays common to all the cell types to provide an easy-to-use annotation for a wide variety of circumstances. However, a number of particular applications may require specialized annotations tailored to specific data contexts and questions (e.g., investigation of nuclear architecture or systems biology). The current ENCODE release, in fact, provides a data-rich context for a subset of cell types. Deep integration over many advanced assays allows us to connect many regulators and non-coding elements into multi-modal networks, including proximal and distal ones, such as TF and RNA-binding proteins (RBP) to gene, enhancers to gene, and TF-to-enhancer-to-gene. Here, focusing on these data-rich cell types, we developed an integrative and network-associated annotation, which may serve as a valuable resource for cancer genomics.

Cancer genomics is, in fact, one of the best applications to illustrate many key aspects of ENCODE. Unlike many other diseases, cancer is very much a disease of whole-genome alteration and dysregulation^9–12^. Moreover, cancer cells usually display aberrant behavior of key regulators, extensive epigenetic remodeling, and apparent transitions between cell states^13–17^. Finally, the systems aspect of cancer has been extensively studied, providing a need to connect linear genome annotation with pathways and networks^18–24^.

In the following sections, we first introduce the resource. We then demonstrate its utility through several applications such as evaluation of regulator activity, regulatory network rewiring, investigation of tumor-to-normal cell-state trajectories, and interpretation of expression and mutation profiles using extended genes. Synthesizing these, we propose a framework to prioritize regulators, elements, and nucleotides and then perform targeted experimental validations using different techniques.

## Results

### The ENCODEC resource

ENCODEC is a specialized *ENCODE* companion resource for *C*ancer genomics. First, using the ENCODE data, for each cancer, we try to find the best tumor-normal pairing available. To achieve this, we often constructed a composite normal by reconciling multiple related cell types (see Supplementary section 1.4). Although the pairings are only approximate, many of them have been widely used in prior studies (see Supplementary section 1.3). Then we build a derived resource. Overall, this consists of (1) comprehensive networks that allow us to see global alterations in network rewiring and regulatory hierarchy; (2) an annotated catalog of cell types that allows us to place oncogenic changes relative to normal and stem cells; and (3) compact noncoding annotations and extended gene definitions that can potentially increase statistical power to interpret genome variation (both germline and somatic) and gene expression changes. Practically, the resource consists of a set of annotation files and computer codes available online (ENCODEC.encodeproject.org).

**Fig. 1:**
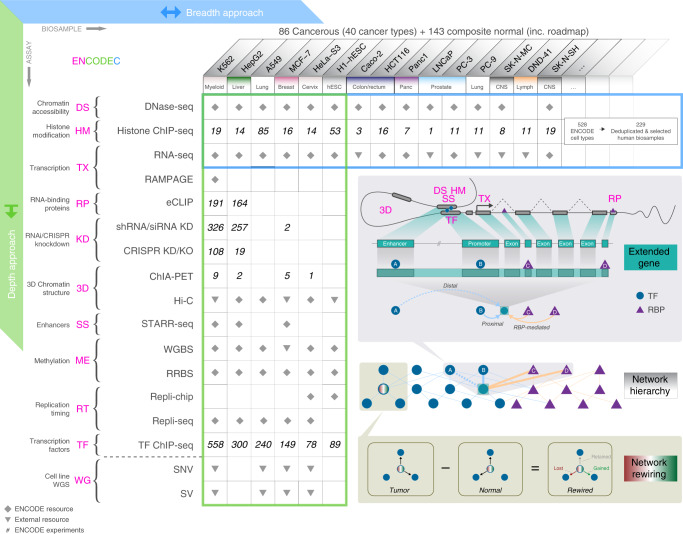
Overview of the ENCODEC resource. Table columns list cell types and rows list assays. Blue table boundary: Cell types with assays in the ENCODE Encyclopedia highlight the breadth of the resource. The large number of cell types allows for comparative analyses between cell-types, as well as cell-type specific analyses. Green table boundary: cell-type specific analyses based on deep annotations of cell lines. The integration of assays allows for high-resolution investigation of genomic biology. Inset: we use annotations from cell-type specific ENCODE assays to build extended gene definitions—coding and non-coding elements that are linked according to their interaction and associated function (top). We relate transcription factors (TFs) and RNA binding proteins (RBPs) in a joint network hierarchy that describes their regulatory potential (middle). By comparing regulatory networks in tumor and normal ENCODE samples, we develop rewiring networks that may relate to regulatory changes that occur in the context of normal-to-tumor transition (bottom).

Figure 1 illustrates two key dimensions of the resource and the ENCODE data: breadth across cell types and depth across assays. From the depth of the ENCODE experiments in data-rich cell types, we constructed a deep, integrated annotation with two key characteristics: (1) noncoding elements are compactly defined to more precisely locate functional sites, and (2) these discontinuous regulatory regions are linked to genes to form extended-gene definitions. Extended genes are highly dynamic and may change considerably across cell types (similar in fashion to cell-type specific isoforms for conventional gene structures).

In particular, to define distal regulatory elements (e.g. putative enhancers), we integrated up to 10 histone modification ChIP-seq experiments per cell type using a support vector machine approach^25^. This procedure uses a shape-matching filter to predict enhancers based on element-associated meta-profiles of epigenetic features^26^. It has been extensively validated, giving an overall error rate of ~20% at 80% sensitivity (see Supplementary section 2.1.2.1). Next, where possible, we intersected these regions with positives called from STARR-seq experiments (see Supplementary section 2.1.2.2). This resulted in a substantially shorter list of distal elements than one gets with conventional approaches. Further, we restricted individual annotated elements down to a core definition enriched for functional sites by pruning based on binding motifs and using novel advanced assays such as eCLIP^27^. As a result, our annotations are short in length but have a high degree of conservation (see Supplementary section 2.4.1).

Thus, overall, our annotation is compact in two respects: it contains fewer total elements (because the deep integration across many assays removes many potential false positives) and each individual element tends to be shorter in length yet is more enriched in functionally relevant nucleotides. In principle, both these facts benefit statistical power through decreasing multiple testing burden or more sharply defining core regions by removing nonfunctional nucleotides in each element.

We also linked together the above compact annotation elements to define extended gene structures, which may also increase power in many circumstances (see Supplementary section 2.6). Diagramed in Fig. 1, the extended gene links the non-coding promoters and enhancers to genes. To define enhancer-gene linkages, we first used physically based linkages from Hi-C. These are accurate but often with fairly low-resolution, potentially spuriously connecting genes within the same topologically associating domain (TAD). Therefore, we pruned this with activity correlations: we correlated the chromatin marks on enhancers and gene expression on potential targets (both within the same TAD) using a machine learning approach^28^, to generate a high-confidence subset (see Supplementary section 2.2). The extended gene annotation potentially enriches the number of functional sites being tested, thus increasing power. Second, it helps with the interpretation of noncoding elements by linking them to genes. Third, it allows us to subset non-coding annotations by the many well-known gene categories, for instance, cancer-associated and metabolic genes.

Building on the extended gene annotation, we constructed detailed networks linking regulators to genomic elements to target genes. Specifically, we built both distal and proximal networks linking TFs to genes. This was accomplished by directly inferring from ChIP-seq experiments either by TF-promoter binding or indirectly via TF-enhancer-gene interactions in each cell type (see details in Supplementary section 2.2). We then pruned the full networks to just the strongest interactions using a signal shape algorithm that keeps the most-relevant peaks by weighting their location by the expected binding profile of each TF^29^ (details in Supplementary section 2.3.3). Similarly, we also defined an RBP network from eCLIP experiments. For the data-rich cell types with numerous TF ChIP-seq experiments, we further built cell-type specific regulatory networks and then compared these between matched tumor and normal cell types, enabling measurement of the change in connections during oncogenesis (i.e., network rewiring). Compared to other network definitions (e.g. via imputation based on motifs^30^), our ENCODE TF and RBP networks are based on direct experimental evidence and can capture more literature-supported regulations and correlate better with knockdown experiments (see Supplementary section 2.4.4).

### Leveraging ENCODE networks to prioritize regulators

**Fig. 2:**
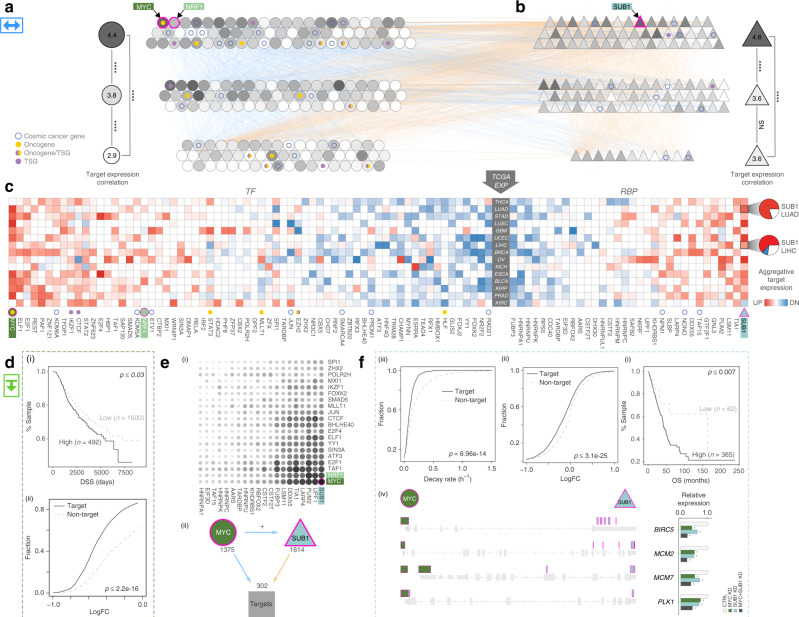
Regulatory network hierarchies. **a** TFs and **b** RBPs are systematically organized into a hierarchy, forming a joint TF-RBP regulatory network. Higher layer elements tend to regulate lower layer elements. **c** The regulatory potentials of TFs/RBPs to drive tumor-to-normal expression changes are shown as a heatmap; red and blue indicate up- and down-regulation respectively. **d** Elevated MYC regulatory activity is associated with reduced disease-specific survival (DSS) in breast cancer (i); MYC knockdown in MCF-7 leads to significantly larger expression reduction in MYC target genes (ii). **e** MYC expression is more positively correlated with its target genes as compared to other TFs (top); MYC frequently forms FFLs with NRF1. These are mostly coherent FFLs and OR-gate logic predominates (bottom). **f** Elevated SUB1 regulation activity is associated with reduced overall survival (OS) in lung cancer (i); SUB1 knockdown in HepG2 leads to reduced target gene expression (ii); Targets of SUB1 show slower mRNA decay rate (iii); for cancer-associated target genes of MYC and SUB1, gene expression is decreased with both MYC and SUB1 knockdown (KD), compared with knockdown of either MYC or SUB1 individually, and compared to control (iv).

After constructing the multi-modal TF-RBP network, we systematically arranged it into a hierarchy (Fig. 2a, b). Here, regulators are placed at different levels such that those in the middle tend to regulate those below them and, in turn, are more regulated by regulators above them (see Supplementary section 3.1). In the hierarchy, we find that top-layer TFs and RBPs more significantly drive differential expression (*p*-value < 2.2e-16, one-sided Wilcoxon Test). The joint TF-RBP networks also enable investigation of cross-regulation between TFs and RBPs. Interestingly, we find that there are fewer TF-RBP interactions on the bottom level, as compared to top and middle-level ones (*p*-value = 3.4e-16 and 1.2e-09, one-sided Wilcoxon Test, see Supplementary section 3.7). Furthermore, we notice a well-known oncogene MYC is one of the master TFs that sits on the top-level of the hierarchy. Interestingly, MYC not only directly regulates the expression of other TFs but also targets many RBPs.

Our networks also enable gene-expression analyses in tumor samples. We used a regression-based approach to systematically search for the TFs and RBPs most strongly driving tumor-normal differential expression across different cancers (see Supplementary section 3.4). For each patient, we tested the degree to which a regulator’s activity correlates with its target’s tumor-to-normal expression changes. We then calculated the percentage of patients with these relationships in each cancer type and presented the overall trends for TFs and RBPs in Fig. 2c. As expected, we find that the target genes of MYC are significantly up-regulated in numerous cancer types—in fact, it has the most up-regulated targets of any TF—consistent with its well-known role as a key oncogenic TF^31,32^. We further validated MYC’s regulatory effects using knockdowns (Fig. 2d). Consistent with our predictions, the expression of MYC targets is significantly reduced after MYC knockdown in MCF-7 (Fig. 2d).

**Fig. 3:**
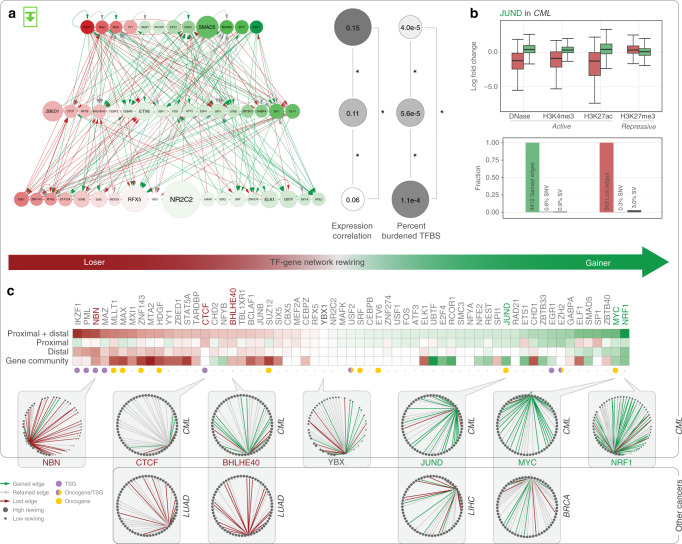
TF-Gene network rewiring. Green and red arrows designate edge gain and loss, respectively. **a** Cell-type specific network using K562 and GM12878: top layer TFs significantly drive tumor-normal differential expression; bottom layer TFs are more often associated with burdened binding sites. **b** JUND is a top edge-gainer in CML, and its targets demonstrate increased gene expression. However, few of its binding sites are affected by SVs or SNVs. **c** Rewiring index in CML by direct edge counts using both proximal and distal networks (top) and by gene community analysis (bottom). Comparisons to TF-gene rewiring networks in other cancers are also shown.

We analyzed the RBP network in a manner similar to the TF network, finding regulators associated with each cancer. For example, the ENCODE eCLIP profile for the RBP SUB1 has binding peaks enriched on the 3′UTR regions of genes, and the predicted targets of SUB1 were significantly up-regulated in many cancer types (Fig. 3f, left). As an RBP, SUB1 has not been associated with cancer previously, so we sought to investigate its role. Knocking down SUB1 in HepG2 cells significantly down-regulated its targets, and the decay rate of SUB1 targets is lower than those of non-targets (Fig. 3f, right). Moreover, we find that up-regulation of SUB1 targets may lead to decreased patient survival in some cancer types.

We then used the regulatory network to investigate how prioritized regulators interact with each other and other genes. For TFs, we first looked at how MYC’s target genes are co-regulated by a second TF. An accounting of all the possible three-way co-regulatory relationships is shown in Fig. 2e. We find that the most common pattern is the well-characterized feed-forward loop (FFL). In this case, MYC regulates both another TF and a common target of both MYC and that TF. Many of the FFLs involve well-known MYC partners such as MAX and MXL1. However, we also discovered many involving NRF1. Upon further examination, we find that that the MYC-NRF1 FFL relationships were mostly coherent, i.e., amplifying in nature (see Supplementary section 3.7). We further studied the FFLs by organizing them into logic gates, in which two TFs act as inputs and the target gene expression represents the output^33^. We find that most of these gates follow either an OR or MYC-always-dominant logic, very much in consonance with MYC’s role in driving oncogenesis.

Similarly, with respect to RBPs, we find that the top co-regulatory partner of SUB1 is, in fact, MYC. SUB1 is a direct target of MYC in many cell types (see Supplementary section 3.7) and also forms many FFLs with MYC in the regulatory network. We hypothesized that MYC binds to the promoter regions of key oncogenes to initiate their transcription, whereas SUB1 binds to their 3′UTRs to stabilize their RNA transcripts. Such collaboration between MYC and SUB1 potentially could result in the overexpression of several key oncogenes (see Supplementary section 3.7). To validate this hypothesis, we knocked down MYC and SUB1 in HepG2 and used qPCR to quantify changes in gene expression. As expected, the expression of oncogenes (such as MCM2, MCM7, BIRC5, and PLK1) is significantly reduced (Fig. 2f and see Supplementary section 3.5).

### Measuring network rewiring

In addition to the TF regulatory activity change through expression analysis above, we also directly measured the fractional number of regulatory edge changes for tumor-normal pairs, to study how TF targets change in oncogenesis. We call this the rewiring index and ranked TFs according to it (Fig. 3c). In leukemia, well-known oncogenes (such as MYC and NRF1) were among the top edge gainers, while the well-known tumor suppressor IKZF1 is the most significant edge loser (Fig. 3c). Mutations in IKZF1, in fact, serve as a hallmark of various forms of high-risk leukemia^34,35^. We observed a similar rewiring trend using distal, proximal, and combined networks (Fig. 3c). This trend was also consistent across a number of cancers: in particular, highly rewired TFs such as BHLHE40, JUND, and MYC behaved similarly in lung, liver, and breast cancers (Fig. 3c).

In addition to direct TF-to-gene conne7ctions, we also measured rewiring using a gene-community model. Here, the targets within the regulatory network were characterized in terms of self-consistent modules of related genes (so-called gene communities). Instead of directly measuring the changes in a TF’s targets between tumor and normal cells, we determined the changes in regulated gene communities (via a mixed-membership model, see Supplementary section 4.3.3). Similar patterns to direct rewiring were observed (Fig. 3c).

Overall, we find that the majority of rewiring events were associated with notable gene-expression and chromatin-status changes, but not necessarily with direct variant-induced motif loss or gain events (Fig. 3b). For example, JUND is a top edge gainer in K562. Most of its gained targets in tumor cells demonstrate higher levels of gene expression, stronger active and weaker repressive histone-modification signals, yet few of its binding sites are mutated, either by SNVs or SVs. This is consistent with previous work^36^, and with a few notable exceptions, we find a similar trend for the rewiring events associated with JUND in liver cancer and, largely, for other factors in a variety of cancers (see Supplementary section 4.4).

We also organized the cell-type specific networks into hierarchies, as shown in Fig. 3a (similar to the universal, cross-cell-type hierarchies described earlier in Fig. 2a, b). We find that the strongest edge gainers and losers, driving the rewiring of the regulatory network, sit at the top level of these hierarchies in blood cancer. In addition, we find the TFs more associated with driving cancer gene expression changes also tend to be at the top. MYC is a most prominent example of both a highly rewired TF and one driving expression. In contrast, the more mutationally affected TFs sit at the bottom of the hierarchy. To some degree, this is consistent with our results in Fig. 3b showing that binding site mutations do not drive the regulatory change.

### Placing cancer cells in the context of ENCODE biosamples

**Fig. 4:**
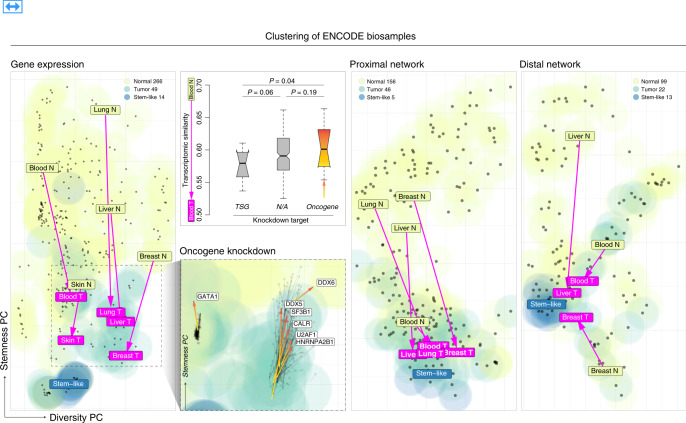
Oncogenic transformation and cell state. We project the expression profiles (left, poly-A long RNA-seq), proximal network (second from right, CTCF ChIP-seq), and distal network (right, candidate cis-regulatory elements) of the ENCODE cell types to a lower dimension space. Stem-like cell types formed a cluster, suggesting stem-like cell types have a distinct profile from normal and cancerous cell types. Further, we find that cancerous cell types tend to locate closer to stem-like clusters. Oncogene knockdown in K562 led to more transcriptomic similarity to a normal cell-type, and tumor suppressor gene (TSG) knockdown led to greater similarity to a tumor cell-type (second from left, top, in comparison to GM12878). In general, we find that oncogene knockdown leads to a slight reversion towards normal state along the stem-like component (second from left, bottom).

ENCODE data provides an additional way of studying the oncogenic transformation beyond network rewiring: via placing various cancer cells in a context of many cell types (in cell space). This is possible because of the wide variety of cell types profiled in the new ENCODE release, which includes many stem cells, especially the data-rich H1 cell line. We are particularly interested in comparisons to stem cells since a decades-old paradigm has held that at least a subpopulation of tumor cells can self-renew, differentiate, and regenerate in a manner similar to stem cells^37–42^. For such comparison, we first projected the RNA-seq data from 299 ENCODE cell types into a low-dimensional space (using the procedure described in Li et al.^43^, see Supplementary section 5.1). We find that various types of stem cells form a tight cluster (Fig. 4). Moreover, there is a trend where the trajectory from normal to tumor cells involves moving toward stem cells, along a single stem-like component. This is true for a variety of different cancers. This observation is consistent with previous efforts using expression and methylation analysis^44^. Notably, we observed a consistent (or even stronger) pattern from proximal and distal chromatin data, which can be viewed as the underlying cause of the observed gene expression changes.

It is well-known that dysregulation of oncogene TFs is a hallmark of tumor progression^11,45–48^. Key genes, such as MYC, initiate overexpression of other oncogenes in tumor cells^32,49^. We can use the cell-space diagram to see the degree to which these TFs contribute to the state of cell differentiation: in particular, we measured the perturbations induced by oncogenic TFs through expression comparisons before and after TF knockdowns. Interestingly, the expression profiles usually reverted slightly back towards normal state upon oncogene knockdown, along the stem-like component. One can see this difference more precisely and test it statistically if one restricts just to the single transition between GM12878 and K562 (Fig. 4).

### The extended gene representation

After identifying key regulators, we next aimed to prioritize their associated genomic elements. To do this, we combined the extended gene annotation with expression and mutation data from patients. We show three examples where this is useful.

**Fig. 5:**
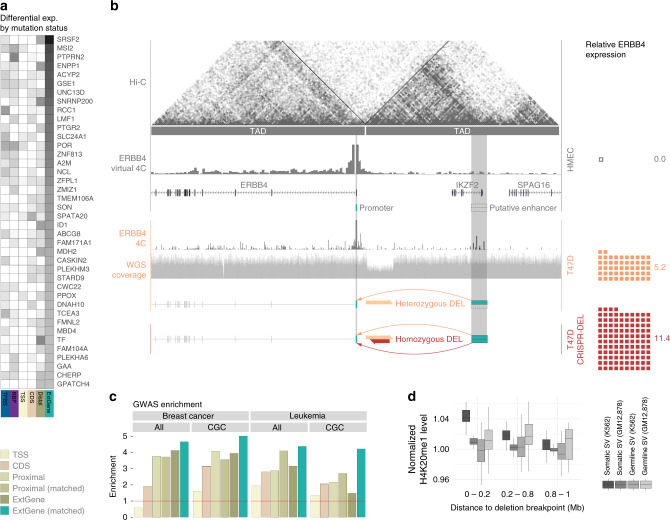
Extended genes and mutation burden analysis. **a** Mutation status in extended genes can explain expression differences for a larger number of genes than other annotations, such as annotations of coding sequences (CDS). **b** A 130-kbp deletion in the breast cancer cell line T47D potentially links a distal enhancer to the promoter of ERBB4, leading to its activation. This change does not affect coding sequences, highlighting the value of an extended gene annotation. **c** Cancer-associated GWAS SNVs display greater enrichment with the inclusion of proximal and distal annotations in extended gene definitions. **d** Somatic structural variant breakpoints in K562 tend to be associated with the activating histone mark H4K20me1, but not in GM12878.

First, our extended gene definitions can be used for associating differential expression with mutational status. For example, we combined the mutation and expression profiles from large cohorts, such as those in TCGA, and found that mutation status in extended genes can better explain the tumor expression than other annotations, such as just canonical coding sequences (CDS). That is, one can much better predict tumor-normal differential expression from mutations in the extended gene as compared to just in CDS or in individual promoters or enhancers (see Supplementary section 6.1). One example of the explanatory potential of the extended gene is seen for the splicing factor SRSF2, which has been shown to affect liver cancer progression and for which differential expression in HepG2 can be well predicted using mutations in the extended gene (Fig. 5a, *p*-value = 0.002, one-sided Wilcoxon test).

The second example is cancer genome-wide association study (GWAS) variant enrichment. That is, the enrichment of cancer-associated GWAS germline SNPs in particular genome regions. The enrichment significantly increases in going from CDS to extended genes for both breast cancer and leukemia (Fig. 5c). This trend is much more pronounced when the newly added non-coding annotations are from matched cell types. One may further subset the genes according to different subcategories associated with cancer and identify enrichment. For instance, we observed a significant enrichment in genes from the Cancer Gene Consensus (CGC) in breast cancer based on the extended gene annotation. This sub-setting by well-known gene categories is not possible using conventional non-coding annotations.

One can get a physical sense of the importance of the extended gene by looking at a situation where a genomic variant rearranges the extended gene structure without affecting the coding regions. We find such an example in the breast cancer cell line T47D, where a 130-kbp heterozygous deletion links a distal enhancer to the ERBB4 promoter and results in the activation of this well-known oncogene^50,51^ (Fig. 5b). The enhancer is not connected to ERBB4 in normal breast tissue; however, in T47D, the deletion, located around 45 kbp downstream from the ERBB4 promoter, merges two Hi-C TADs in an allele-specific way. We tested this through CRISPR editing, by excising an 86 bp sequence within the wild-type allele of the heterozygous deletion containing the CTCF binding sites at the boundary of the two TADs. This CRISPR excision confirmed the elevated ERBB4 expression (see Supplementary section 6.4).

Another perspective on the effect of SVs changing chromatin structure is provided from broadly surveying SVs in a number of the data-rich ENCODE cells types. (Note, ENCODE provides SV call sets based on integration of assays including Hi–C for a number of these cell lines, see Supplementary section 6.5.3). In particular, in Fig. 5d, we surveyed regions around somatic SV breakpoints in K562. We find that the activating histone mark H4K20me1 occurs preferentially around these breakpoints. This enrichment was not observed using GM12878 histone mark data at these exact same locations. We further examined the GM12878 H4K20me1 levels proximal to germline breakpoints (for common variants as determined from the 1000 Genomes Project^52^) and also find no enrichment (see Supplementary section 6.5). One potential implication is that the somatic SVs in tumor cells may be associated with creating active regions of chromatin.

### Step-wise prioritization framework

**Fig. 6:**
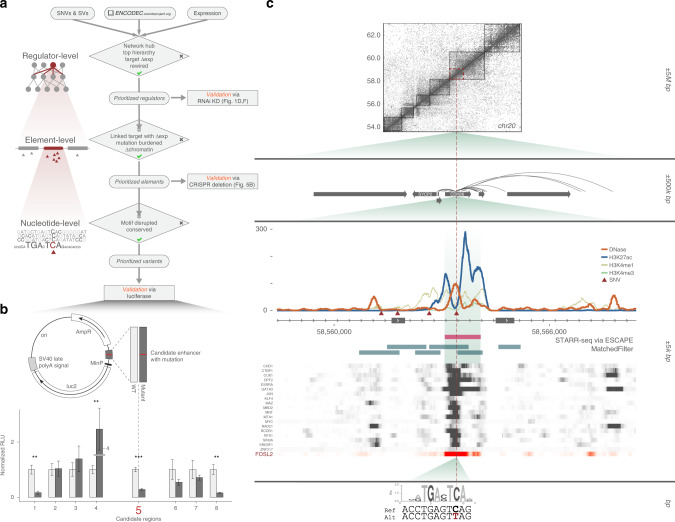
Variant prioritization and validation. **a** A stepwise prioritization scheme for genomic regulators, elements, and variants, using the ENCODEC resources. At each step of prioritization, we indicate criteria for prioritization, as well as the applicable validation assay. **b** Small-scale validation of prioritized variants using a luciferase reporter assay. Candidate region 5 showed the most significant degree of differential expression and was selected for follow-up analysis. **c** Multiscale integrative analysis of candidate region 5 with assorted functional genomics data. The affected region is observed in the context of large-scale Hi–C linkages (top), as well as element-level signal tracks of histone modification marks and DNase hypersensitivity together with various TF binding events (middle), and nucleotide level disruption of the FOSL2 motif (bottom).

Collectively, as described in Fig. 6, ENCODEC enables a step-wise prioritization that allows us to pinpoint key regulators, noncoding elements, and variants associated with oncogenesis. Specifically, we first highlighted regulators that are either greatly rewired, located in hubs, sit at the top of the hierarchy, or significantly drive expression changes in cancer. We then prioritize functional elements associated with these regulators that are either highly burdened by mutations, undergo large chromatin changes, or change in extended gene linkages. Finally, on a nucleotide level, we prioritize SNVs by estimating their ability to disrupt or introduce specific binding sites and assessing to what degree they lie in a prioritized element.

We instantiated our prioritization workflow in a few select cancers and experimentally validated the results. In particular, as described above, we subjected some key regulators, such as MYC and SUB1, to knockdown experiments (Fig. 2d, f) and we measured the effect of SVs on element linkages via CRISPR engineered deletions (Fig. 5b). Finally, we selected key SNVs based on their disruption of enhancers with a strong influence on gene expression. These SNVs were prioritized based on element-level mutation recurrence in breast-cancer cohorts, as well as motif disruption scores. Of the eight motif-disrupting SNVs that we tested, six exhibited consistent up- or down-regulation relative to the wild-type in multiple biological replicates (see Supplementary sections 7.2 and 7.3).

One particularly interesting example occurs in an intronic region of CDH26 in chromosome 20 (Fig. 6c). The signal shapes for both histone modification and chromatin accessibility (DNase-seq) data indicate its active regulatory role as an enhancer in MCF-7. This was further confirmed by STARR-seq (Fig. 6c). Hi-C and ChIA-PET linkages indicated that the region is within a TAD and validated a regulatory connection to the cancer-associated gene SYCP2^53^. We further observed strong binding of many TFs in this region in MCF-7. Motif analysis predicts that a common mutation in breast cancer affects this region, and significantly disrupts the local binding affinity of several TFs, such as FOSL2 (Fig. 6c). Luciferase assays demonstrated that this mutation introduces a 3.6-fold reduction in expression relative to the wild-type, indicating a strong repressive effect on enhancer functionality.

## Discussion

In this paper, we describe a customized ENCODE annotation: a companion resource providing an integrative network annotation including extended gene. Cancer genomics is an ideal application to highlight the value of the resource, and we show how it can help describe oncogenic transformations in terms of cell-space trajectories and network rewiring. We also use the specialized annotation to prioritize key regulators, element, and variants.

There remain several caveats associated with our resource. First and most obviously, proper somatic variant annotation and, especially driver discovery, is a multiple-step process that requires coordinated, large-scale effort. Extensive follow-up validations are required, in addition to the careful calibration required for statistical identification of mutation recurrence and the many biases in sequencing (e.g. taking into account the elevated mutation rate associated with TF binding sites^2,6^, sequence coverage and mutational signatures^54,55^). While we hope that ENCODE data and annotation can be useful in this context, they are not sufficient. Second, our resource associates cancer types with ENCODE cell lines and then secondarily pairs them with a composite normal. Both types of pairings are, by nature, approximate. Tumor cells from a given patient show distinct molecular, morphological, and genetic profiles^56–59^. Moreover, linking cancer to one specific cell-type may not even fully capture the heterogeneity seen in actual tumors^60^. In the future, technological advances, such as single-cell sequencing, may allow cell-type or tissue-type comparisons at a higher resolution^61–65^. Nevertheless, we feel that our annotation and networks currently provide the best available view of the regulatory changes in oncogenesis.

Finally, we argue here that, somewhat counter-intuitively, a comprehensive non-coding annotation that, in the extreme, attempts to assign functional impact to every base in the genome may not always be best suited to specific disease-oriented studies. Rather, the most useful annotation often has several characteristics. First, it is useful to be as compact as possible, both in terms of the extent of individual annotation blocks and in the number of elements. Second, since the currently discovered high impact variants tend to be tightly associated with genes, an optimum non-coding annotation is best invisible, folding itself into gene annotation for better variant interpretation. Third, the network aspect is often needed to allow larger-scale systems perspective. This is particularly valuable for appreciating the overall cellular dysregulation in cancer. With the depth and breadth of the ENCODE assays across thousands of cell types, we endeavored here to provide such a customized annotation resource for cancer and demonstrated its value through several showcase applications. We anticipate that the rapid accumulation of functional genomic data will make possible further, potentially even more specialized, annotation resources for future disease studies.

## Methods

See supplementary information for details on methodology.

## Supplementary information

Supplementary Information

## Data Availability

The derived ENCODEC data have been deposited in the supplementary data website at http://encodec.encodeproject.org/. The source data underlying Figs. 1–6 are provided as a flat file in the supplementary data website as well. All the other data supporting the findings of this study are available within the article and its supplementary information files and from the corresponding author upon reasonable request. All of ENCODE data referenced during the study are available in a public repository from the https://www.encodeproject.org/ website. A reporting summary for this article is available as a Supplementary Information file.

## References

[1] Consortium EP (2012). An integrated encyclopedia of DNA elements in the human genome. Nature.

[2] Frigola J (2017). Reduced mutation rate in exons due to differential mismatch repair. Nat. Genet..

[3] Martincorena I (2017). Universal patterns of selection in cancer and somatic tissues. Cell.

[4] Imielinski M, Guo G, Meyerson M (2017). Insertions and deletions target lineage-defining genes in human cancers. Cell.

[5] Nik-Zainal S (2016). Landscape of somatic mutations in 560 breast cancer whole-genome sequences. Nature.

[6] Sabarinathan R, Mularoni L, Deu-Pons J, Gonzalez-Perez A, Lopez-Bigas N (2016). Nucleotide excision repair is impaired by binding of transcription factors to DNA. Nature.

[7] Supek F, Lehner B (2015). Differential DNA mismatch repair underlies mutation rate variation across the human genome. Nature.

[8] Polak P (2015). Cell-of-origin chromatin organization shapes the mutational landscape of cancer. Nature.

[9] Ntziachristos P, Abdel-Wahab O, Aifantis I (2016). Emerging concepts of epigenetic dysregulation in hematological malignancies. Nat. Immunol..

[10] Liu F, Wang L, Perna F, Nimer SD (2016). Beyond transcription factors: how oncogenic signalling reshapes the epigenetic landscape. Nat. Rev. Cancer.

[11] Gonda TJ, Ramsay RG (2015). Directly targeting transcriptional dysregulation in cancer. Nat. Rev. Cancer.

[12] Timp W, Feinberg AP (2013). Cancer as a dysregulated epigenome allowing cellular growth advantage at the expense of the host. Nat. Rev. Cancer.

[13] Baylin SB, Jones PA (2011). A decade of exploring the cancer epigenome—biological and translational implications. Nat. Rev. Cancer.

[14] Polyak K, Weinberg RA (2009). Transitions between epithelial and mesenchymal states: acquisition of malignant and stem cell traits. Nat. Rev. Cancer.

[15] Yu H, Jove R (2004). The STATs of cancer–new molecular targets come of age. Nat. Rev. Cancer.

[16] Darnell JE (2002). Transcription factors as targets for cancer therapy. Nat. Rev. Cancer.

[17] Jones PA, Baylin SB (2002). The fundamental role of epigenetic events in cancer. Nat. Rev. Genet..

[18] Sanchez-Vega F (2018). Oncogenic Signaling Pathways in The Cancer Genome Atlas. Cell.

[19] Garraway LA, Lander ES (2013). Lessons from the cancer genome. Cell.

[20] Vogelstein B (2013). Cancer genome landscapes. Science.

[21] Horn H (2018). NetSig: network-based discovery from cancer genomes. Nat. Methods.

[22] Creixell P (2015). Pathway and network analysis of cancer genomes. Nat. Methods.

[23] Leiserson MD (2015). Pan-cancer network analysis identifies combinations of rare somatic mutations across pathways and protein complexes. Nat. Genet..

[24] Hofree M, Shen JP, Carter H, Gross A, Ideker T (2013). Network-based stratification of tumor mutations. Nat. Methods.

[25] Sethi A., et al. Supervised enhancer prediction with epigenetic pattern recognition and targeted validation. *Nat. Methods*10.1038/s41592-020-0907-8 (2020). 10.1038/s41592-020-0907-8 PMC8073243 32737473

[26] Kundaje A (2012). Ubiquitous heterogeneity and asymmetry of the chromatin environment at regulatory elements. Genome Res..

[27] Van Nostrand EL (2016). Robust transcriptome-wide discovery of RNA-binding protein binding sites with enhanced CLIP (eCLIP). Nat. Methods.

[28] Cao Q (2017). Reconstruction of enhancer-target networks in 935 samples of human primary cells, tissues and cell lines. Nat. Genet..

[29] Cheng C, Min R, Gerstein M (2011). TIP: a probabilistic method for identifying transcription factor target genes from ChIP-seq binding profiles. Bioinformatics.

[30] Neph S (2012). Circuitry and dynamics of human transcription factor regulatory networks. Cell.

[31] McKeown M. R., Bradner J. E. Therapeutic strategies to inhibit MYC. *Cold Spring Harb. Perspect. Med.* **4**, 14266 (2014). 10.1101/cshperspect.a014266 PMC4200208 25274755

[32] Dang CV (2012). MYC on the path to cancer. Cell.

[33] Wang D (2015). Loregic: a method to characterize the cooperative logic of regulatory factors. PLoS Comput. Biol..

[34] Boer JM (2016). Prognostic value of rare IKZF1 deletion in childhood B-cell precursor acute lymphoblastic leukemia: an international collaborative study. Leukemia.

[35] de Rooij JD (2015). Recurrent deletions of IKZF1 in pediatric acute myeloid leukemia. Haematologica.

[36] Farh KK (2015). Genetic and epigenetic fine mapping of causal autoimmune disease variants. Nature.

[37] O’Connor ML (2014). Cancer stem cells: a contentious hypothesis now moving forward. Cancer Lett..

[38] Ge Y (2017). Stem cell lineage infidelity drives wound repair and cancer. Cell.

[39] Fabregat I., Malfettone A., Soukupova J. New insights into the crossroads between EMT and stemness in the context of cancer. *J Clin. Med.* **5**, 37 (2016). 10.3390/jcm5030037 PMC4810108 26985909

[40] Friedmann-Morvinski D, Verma IM (2014). Dedifferentiation and reprogramming: origins of cancer stem cells. EMBO Rep..

[41] Eppert K (2011). Stem cell gene expression programs influence clinical outcome in human leukemia. Nat. Med..

[42] Gentles AJ, Plevritis SK, Majeti R, Alizadeh AA (2010). Association of a leukemic stem cell gene expression signature with clinical outcomes in acute myeloid leukemia. JAMA.

[43] Li H (2017). Reference component analysis of single-cell transcriptomes elucidates cellular heterogeneity in human colorectal tumors. Nat. Genet..

[44] Malta TM (2018). Machine learning identifies stemness features associated with oncogenic dedifferentiation. Cell.

[45] Hanahan D, Weinberg RA (2011). Hallmarks of cancer: the next generation. Cell.

[46] Vicente-Duenas C, Romero-Camarero I, Cobaleda C, Sanchez-Garcia I (2013). Function of oncogenes in cancer development: a changing paradigm. EMBO J..

[47] Santhekadur PK (2012). The transcription factor LSF: a novel oncogene for hepatocellular carcinoma. Am. J. Cancer Res..

[48] Perkins ND (2012). The diverse and complex roles of NF-kappaB subunits in cancer. Nat. Rev. Cancer.

[49] Lin CY (2012). Transcriptional amplification in tumor cells with elevated c-Myc. Cell.

[50] Yu T (2015). MicroRNA-193a-3p and -5p suppress the metastasis of human non-small-cell lung cancer by downregulating the ERBB4/PIK3R3/mTOR/S6K2 signaling pathway. Oncogene.

[51] Sundvall M (2008). Role of ErbB4 in breast cancer. J. Mammary Gland Biol. Neoplasia.

[52] Genomes Project C (2015). A global reference for human genetic variation. Nature.

[53] Masterson L (2015). Deregulation of SYCP2 predicts early stage human papillomavirus-positive oropharyngeal carcinoma: A prospective whole transcriptome analysis. Cancer Sci..

[54] Bailey MH (2018). Comprehensive characterization of cancer driver genes and mutations. Cell.

[55] Rheinbay E (2020). Analyses of non-coding somatic drivers in 2,658 cancer whole genomes. Nature.

[56] Patel AP (2014). Single-cell RNA-seq highlights intratumoral heterogeneity in primary glioblastoma. Science.

[57] Bedard PL, Hansen AR, Ratain MJ, Siu LL (2013). Tumour heterogeneity in the clinic. Nature.

[58] Meacham CE, Morrison SJ (2013). Tumour heterogeneity and cancer cell plasticity. Nature.

[59] Gerlinger M (2012). Intratumor heterogeneity and branched evolution revealed by multiregion sequencing. N. Engl. J. Med..

[60] Visvader JE (2011). Cells of origin in cancer. Nature.

[61] Tirosh I (2016). Dissecting the multicellular ecosystem of metastatic melanoma by single-cell RNA-seq. Science.

[62] Gawad C, Koh W, Quake SR (2016). Single-cell genome sequencing: current state of the science. Nat. Rev. Genet..

[63] Rotem A (2015). Single-cell ChIP-seq reveals cell subpopulations defined by chromatin state. Nat. Biotechnol..

[64] Eirew P (2015). Dynamics of genomic clones in breast cancer patient xenografts at single-cell resolution. Nature.

[65] Wang Y (2014). Clonal evolution in breast cancer revealed by single nucleus genome sequencing. Nature.

